# Supplementary material for: When Chromatin Decondensation Affects Nuclear γH2AX Foci Pattern and Kinetics and Biases the Assessment of DNA Double-Strand Breaks by Immunofluorescence
Source: Biomolecules. 2024 Jun 14;14(6):703. doi: 10.3390/biom14060703 (PMC11201768; doi:10.3390/biom14060703)

When chromatin decondensation affects nuclear γH2AX foci pattern and kinetics and biases the assessment of DNA double-strand breaks by immunofluorescence

Supplementary Data

Figure S1: Representative immunofluorescence images with the human radioresistant 1BR3 fibroblasts at different magnifications. The X100 magnified image represents one microscopy field (containing generally less than 10 nuclei). Each white bar represents 10 µm. A. γH2AX staining. B. DAPI counterstaining.

A

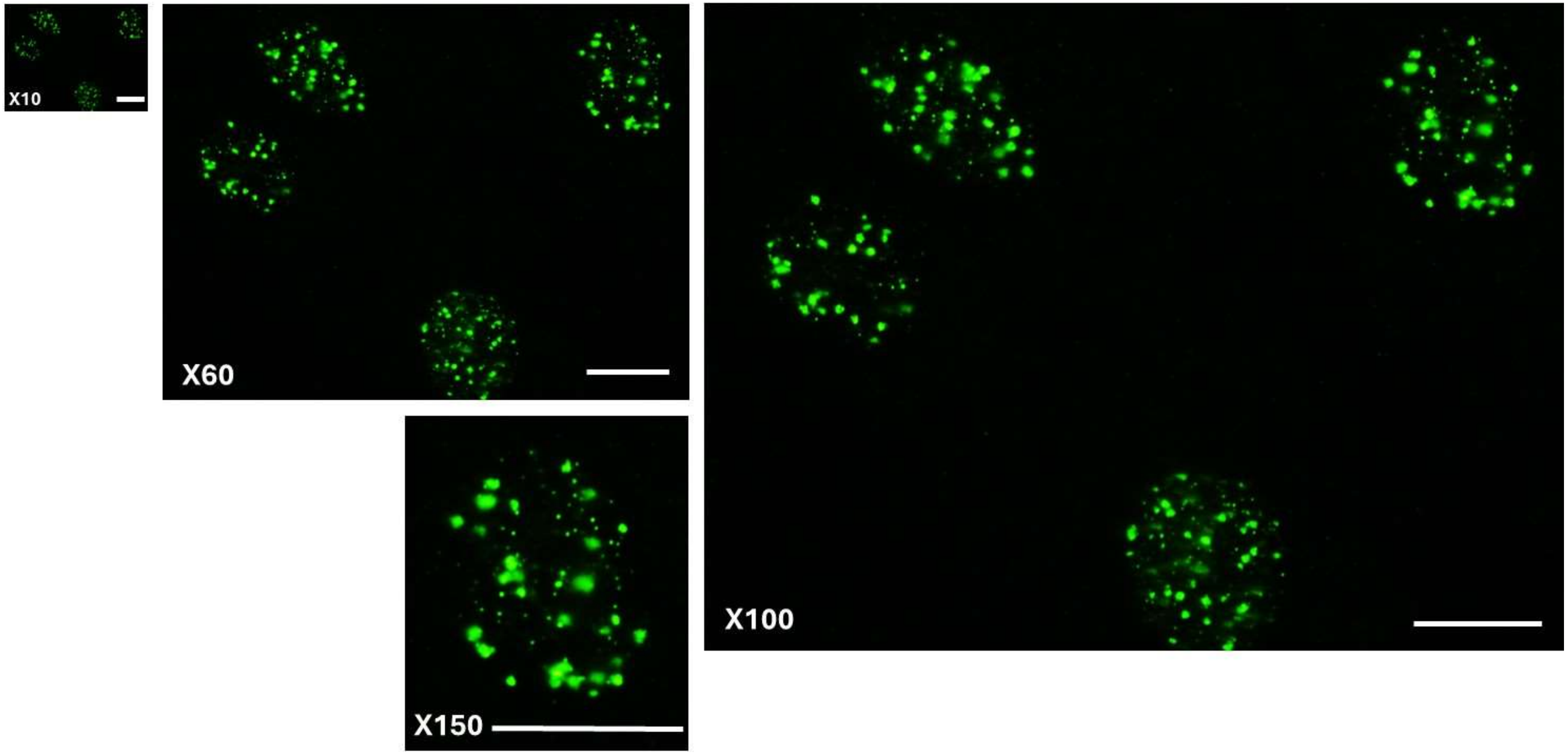

B

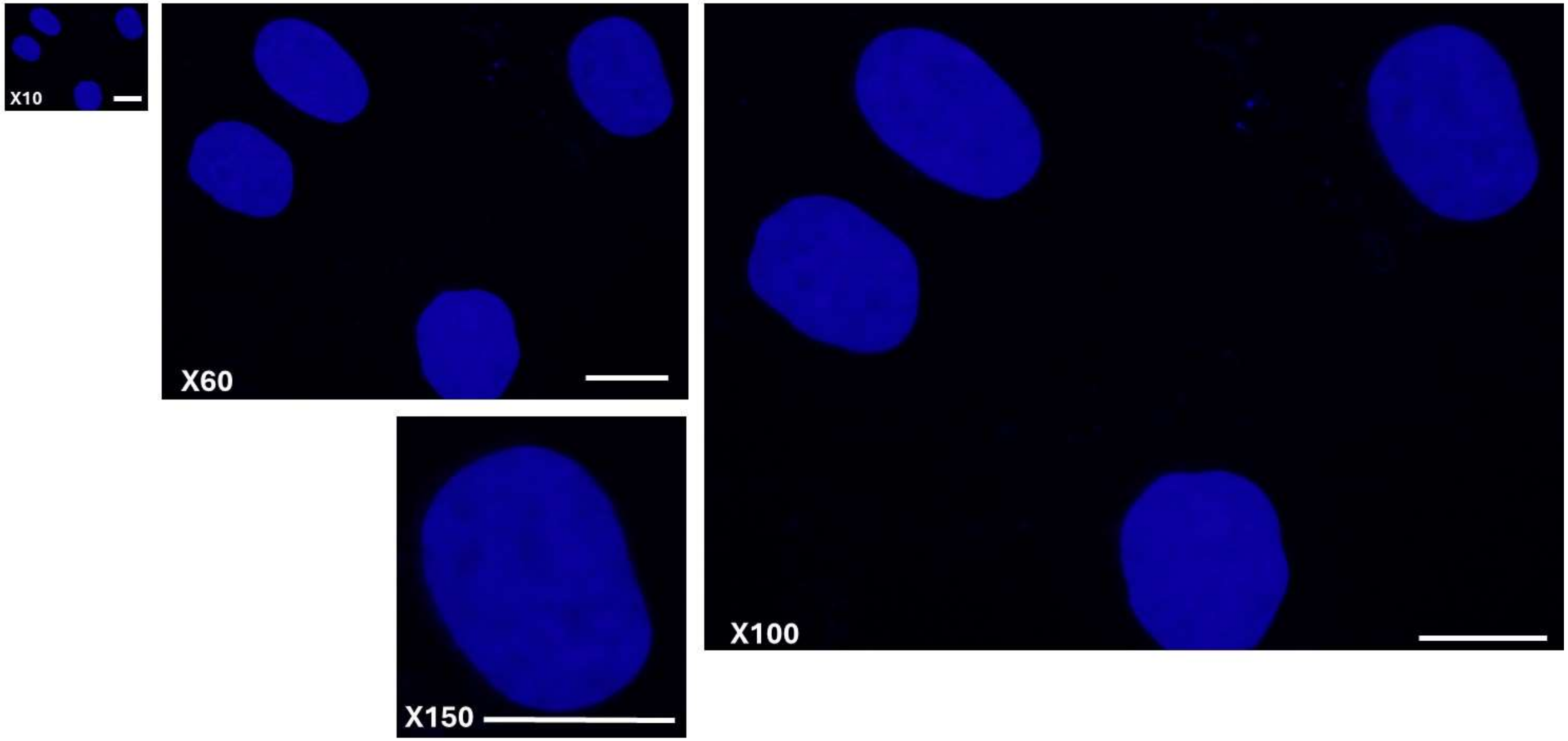

Supplement: Supplementary file 1 [file biomolecules-14-00703-s001.zip › biomolecules-3052362-supplementary.pdf]
